# Psoas hematoma as a rare complication of posterior lumbar interbody fusion: a case report

**DOI:** 10.1186/s12893-020-00942-1

**Published:** 2020-11-11

**Authors:** Bo Deng, Hai Nan Hong, Xin Bing Feng, Zheng Hua Hong, Guo Ping Cai, Dun Hong

**Affiliations:** Department of Orthopedics, Taizhou Hospital of Zhejiang Province, Wenzhou Medical University, Linhai, 317000 China

**Keywords:** Posterior lumbar interbody fusion, Psoas hematoma, Complication

## Abstract

**Background:**

Psoas hematoma rarely occurs in patients with spondylolisthesis who undergo posterior lumbar interbody fusion (PLIF) surgery.

**Case presentation:**

Here we reported a case of a 57-year-old male patient diagnosed with spondylolisthesis who underwent PLIF at the local hospital. Seven days post-surgery, abdominal pain occurred, and the pain in the right lower limb gradually increased. The computerized tomography (CT) indicated a formation of hematoma around the psoas muscle. Digital-subtraction angiography (DSA) suggested a vascular injury, a rupture of the right segmental artery of the lumbar vertebral level 4. The patient then received DSA vascular embolization, after which the lower lumbar segmental artery active bleeding was stopped. One month after discharge, the abdominal hematoma was gradually absorbed, and the pain in the waist, leg, and abdomen disappeared.

**Conclusion:**

Symptoms such as abdominal pain, abdominal distension, and exacerbation of lower limb pain, may suggest the occurrence of psoas hematoma after PLIF. DSA vascular embolization is suggested as the first treatment approach for this type of complication.

## Background

Degenerative lumbar spondylolisthesis is a disorder caused by the slip of one vertebral body over the other. It commonly occurs in people who are over 50 years old and is often associated with lumbar spinal stenosis [1, 2]. Posterior lumbar interbody fusion is widely used in the treatment of lumbar degenerative diseases, such as spondylolisthesis and lumbar spinal stenosis [3, 4]. Yet, this type of surgery is associated with several complications, such as abdominal large blood vessel damage, nerve root injury and epidural hematoma [5, 6]. Kalanithi et al.[7] performed statistical analysis of 66,601 patients with spondylolisthesis who underwent lumbar interbody fusion, reporting that incision hematoma (5.36%), respiratory complications (13.1%), cardiovascular system complications (1.24%), nerve injury (0.80%) and deep vein thrombosis (0.63%) were the most common complications.

Iatrogenic vascular injury rarely occurs after spinal surgery and has an incidence of 0.03% ~ 0.17% [8]. The lower lumbar segmental artery originates from the posterior wall of the abdominal aorta and follows the side of the vertebral body to the posterolateral side. Psoas hematoma occurs secondary to trauma and iatrogenic etiology [9, 10]. If the position of the pedicle screw is poorly placed, the segmental artery can be easily damaged. Here we reported a single case of a patient with psoas hematoma caused by the pedicle screw after PLIF so as to share our experience with complication management related to this condition.

## Case presentation

**Fig. 1:**
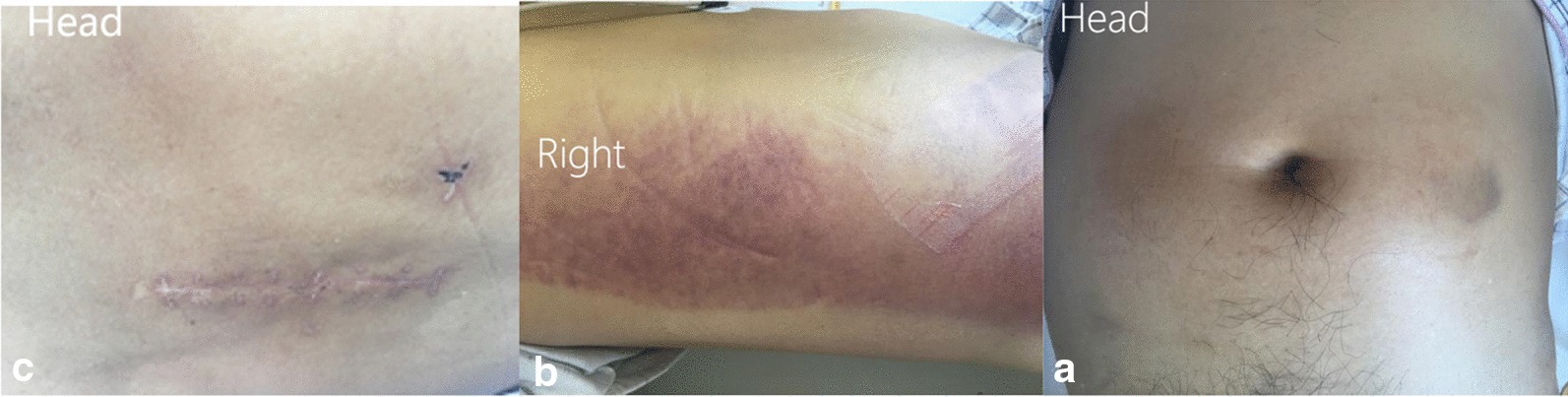
Physical examination 17 days after surgery. **a** Abdominal distension; **b** ecchymosis was observed on the right side of the waist. **c** The incision was healed in the lower back

**Fig. 2:**
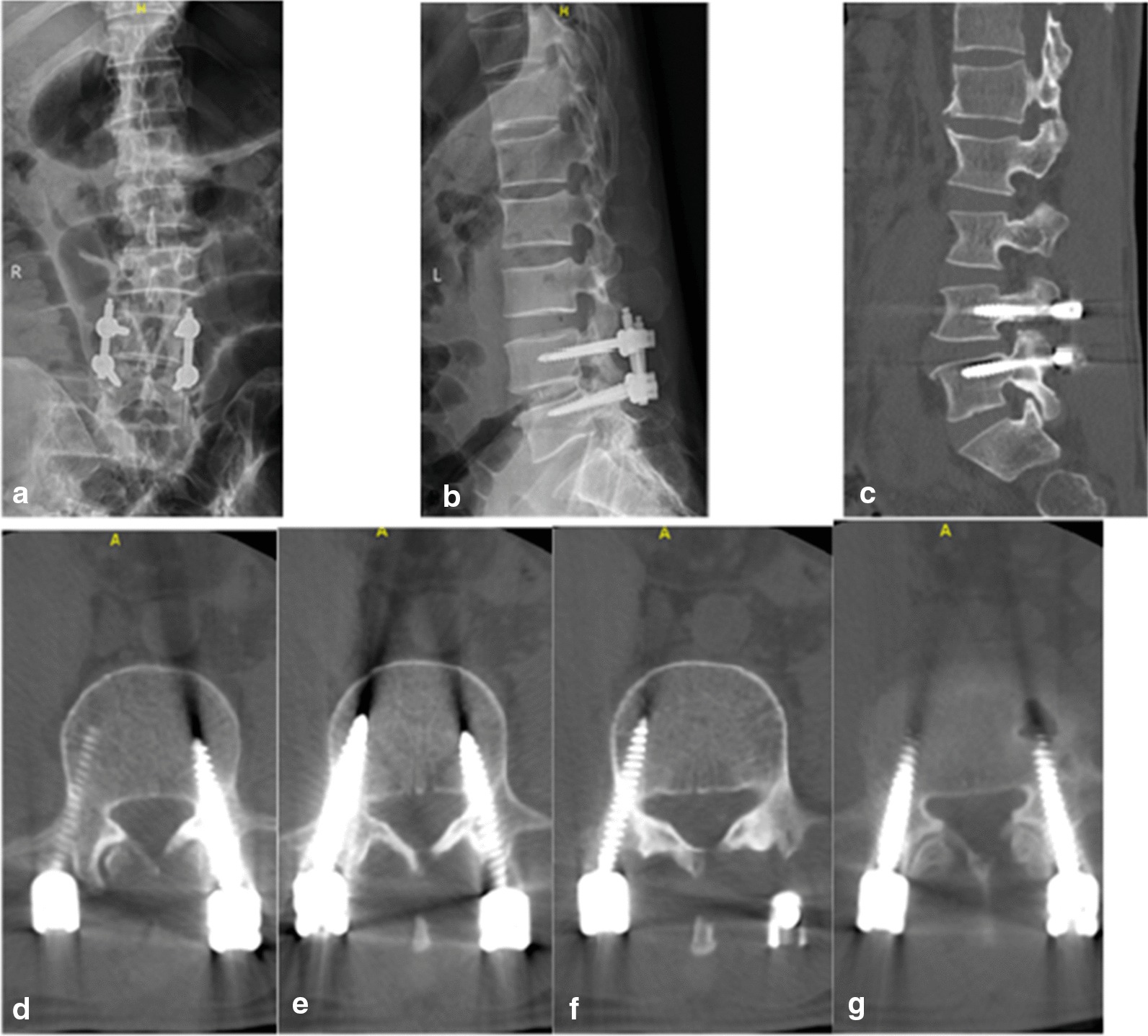
Lumbar CT and X-ray examination 20 days post-surgery. **a**, **b** Anterior lateral radiograph of the patient's lumbar X-ray. **c**–**g** Sagittal and transverse sections of radiograph of the patient's lumbar CT

**Fig. 3:**
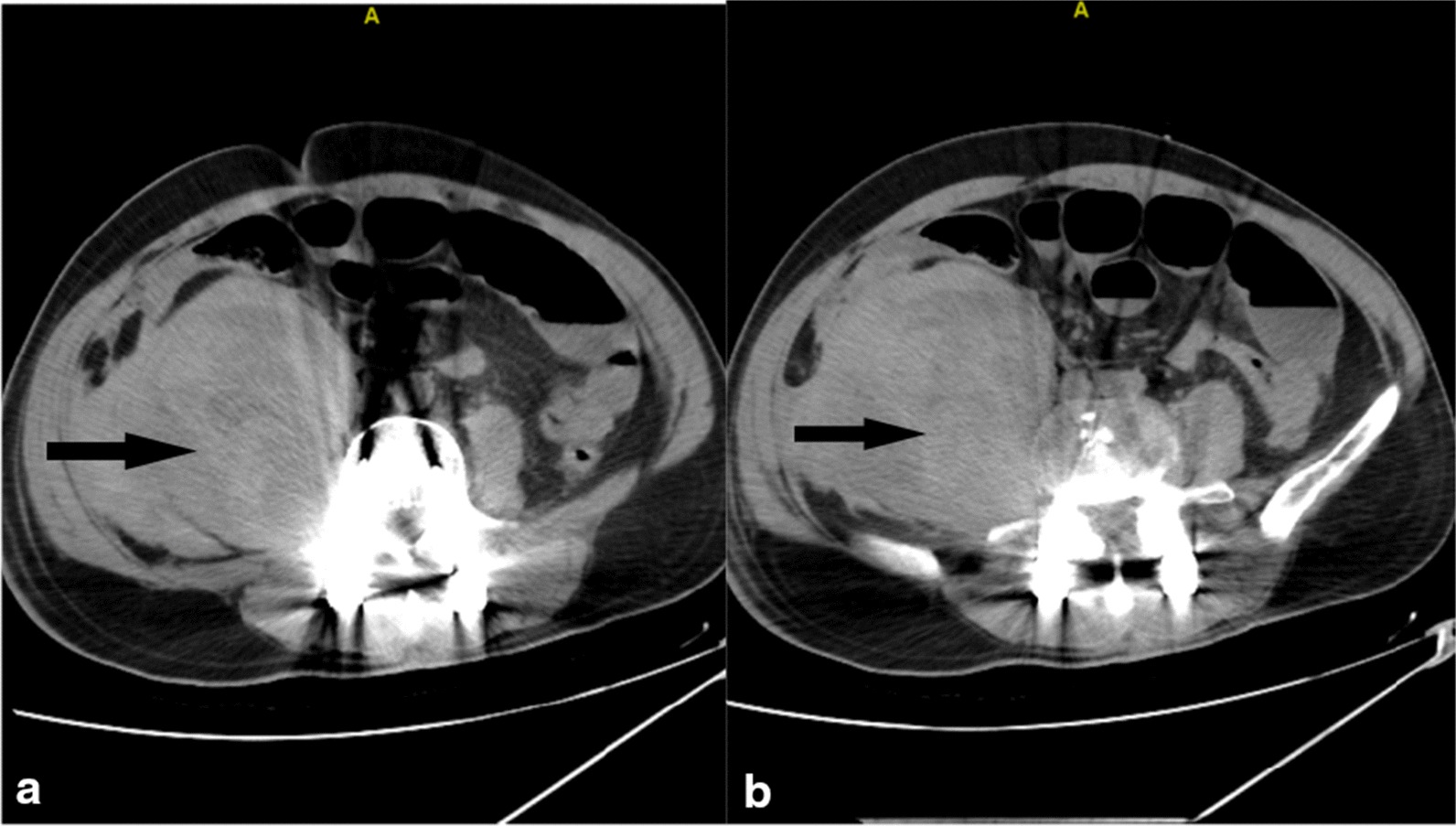
Abdominal CT scan 20 days post- surgery showing prompt psoas hematoma

**Fig. 4:**
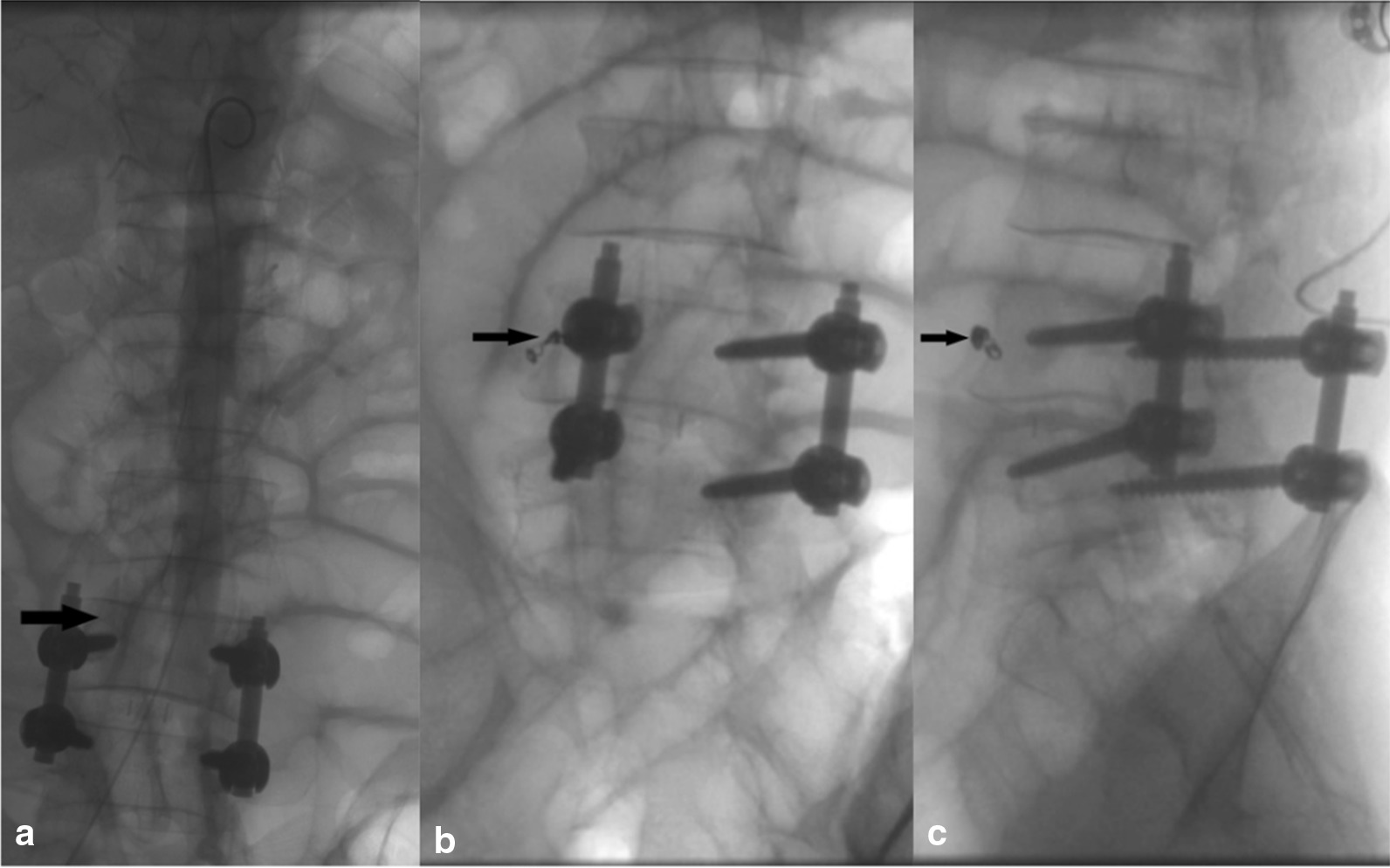
Digital-subtraction angiography (*DSA*). **a** Images showing segmental vessel injury in the right side of the lumbar vertebral body. **b**, **c** The patient underwent lumbar segmental vessel embolization

Herein, we reported a case of a 57-year-old male patient diagnosed with spondylolisthesis who underwent PLIF at the local hospital. On day 7 after the operation, the patient experienced pain in the right lower limb. On day 17, he consulted the hospital due to abdominal pain and severe pain in the right lower limb. Abdominal distension and ecchymosis in the right side of the waist were observed after a physical examination, whereas the incision was healed in the lower back (Fig. **1**). On day 20, the patient underwent further examination. A physical examination of the patient showed a positive right leg elevation test results; yet, the abdominal pain persisted. The patient's lumbar X-ray and lumbar CT showed no complications (Fig. **2**), yet the abdomen CT revealed the formation of psoas hematoma (Fig. **3**). Coagulation tests indicated the following: PT 16.7 s, INR 1.36, APTT 45.8 s, FIB 4.84 g/L, D-dimer 4.61 mg/L; Blood routine indicated the following: WBC 39.8*10^9/L, RBC 1.88*10^9/L, and Hb 55 g/L. The HR was 121 times per minute, Bp82/47 mmHg, which further suggested a hemorrhagic shock. Doctors did not use any hemostatic drugs in the emergency room. In addition, the patient had abdominal tenderness and rebound pain, and thus was immediately treated with 1 million units of vancomycin as prophylactic anti-infective treatment. Moreover, he received 4 IU/mL allogeneic blood transfusion due to anemia symptoms. Subsequently, a massive mass appeared in the abdomen, as well as signs of bruising at the waist; the patient had a history of lumbar fusion surgery. The abdominal purulent infections and intestinal obstruction were then excluded, after which the patient underwent DSA that indicated the injury to the segmental vessel on the right side of the lumbar vertebral body. The patient then underwent lumbar segmental vessel embolization (Fig. **4**).

The patient reviewed blood routine and biochemical tips that suggested a poor function of liver and kidney. The doctor suggested the following: (1) considering that his heart rate was 130 times per minute and the blood pressure was 72/45 mmHg, allogeneic blood transfusion was recommended; (2) the combination of insulin and 50% glucose (Option A) or hemodialysis (Option B) injections were recommended to reduce blood potassium (6.7 mmol/L); (3) renal drugs were recommended for his creatinine levels that were 406 μmoI/L; (4) the patient's ALT was 129μ/ L and AST was 132μ/ L, which indicated poor liver function, condition that should be treated; (5) considering that the abdominal swelling in patient was obvious, and the peristalsis was poor, the enteral nutrition trough nasal feeding tube should be increased; (6) based on the abdominal CT results, puncture hematoma drainage under ultrasound was not recommended.

The patient agreed to follow doctors’ recommendations. After receiving treatment, kidney and liver function improved, blood potassium was reduced (4.3 mmol/L); DSA vascular embolization stopped the lower lumbar segmental artery active bleeding. Consequently, 1 million units of vancomycin were used to prevent potential infection. On day 10 after admission, the WBC was 4.2 * 10^9^/L, and the albumin was 23 g/L. The patient then received 10 g human serum albumin as daily supplementation and the albumin levels returned within the normal range. Consequently, the back and leg pain were gradually reduced, and the ecchymosis on the lower waist disappeared. On day 32 after admission, the patient's waist and leg pain were significantly relieved.

**Fig. 5:**
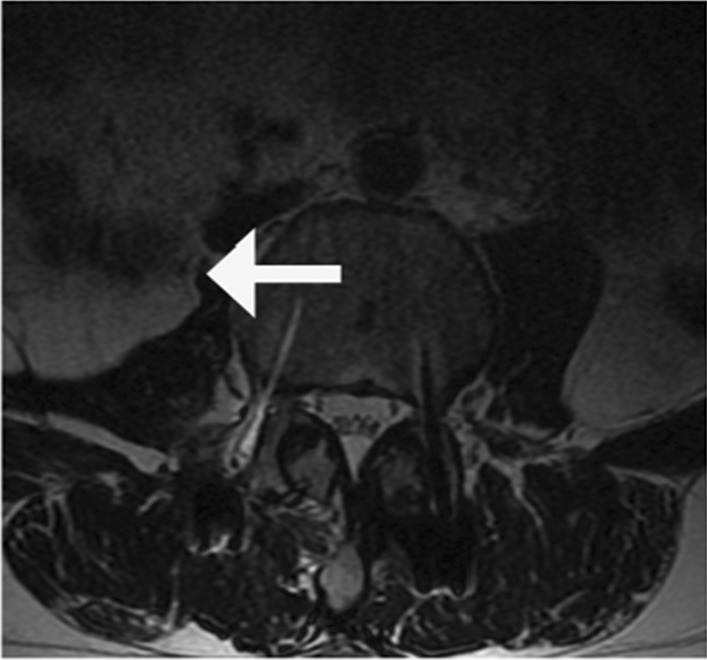
The abdominal MRI showed that the abdominal hematoma was not completed absorbed

At six month after discharge, the patient was reexamined. The abdominal MRI showed that the abdominal hematoma was not completed absorbed (Fig. 5); pain in waist, leg and abdomen disappeared, and the abdominal distension and the right lower limb pain were decreased.

## Discussion and conclusion

PLIF is one of the surgical methods used for the treatment of lumbar degenerative lesions. Over the last decade, the number of PLIF operations has drastically increased in the United State [8]. Yet, this type of surgery is associated with a number of complications, and in some severe cases, may even lead to death.

Iatrogenic vascular injury is a rare complication following PLIF. Previous studies have reported that iatrogenic vascular injury mainly includes iliac arteriovenous, abdominal aorta, inferior vena cava, delayed-type arteriovenous fistula, and delayed abdominal aortic pseudoaneurysm. Its incidence rate is about 0.03% ~ 0.17% [9]. These types of vascular injuries may lead to fatal complications and are mainly caused by surgical errors. When the disc is decompressed, the nucleus pulposus exceeds the leading edge of the vertebral body, placing the interbody cage in the forward direction. Consequently, the front wall of the vertebral body gets worn by the pedicle screw, which in turn may cause injury to the blood vessels. So far, no studies have reported the formation of psoas hematoma in the lower lumbar segmental arterial injury.

The lower lumbar segmental artery originates from the abdominal aorta and follows the lateral side of the vertebral body to the posterolateral side. The lower lumbar segmental artery is posteriorly flanked by a thicker transverse anterior branch of the vertebral body into the psoas muscle. Jaskwhich et al.[11] performed an anatomical study of the posterior and lateral regions of the lumbar disc and found that the segmental vessel distance was 10 ~ 13 mm on the lower edge of the corresponding lower disc. L5 arterial segmental vessel revealed to have the largest variability. Moreover, Ratcliffe [12] suggested that L5 originates from the median sacral artery, while Caglar and colleagues [13] argue that L5 originates from the middle sacral artery, the iliac artery and the L4 segmental artery. Previous literatures reported that the incidence of lumbar segment arterial injury during oblique lumbar interbody fusion(OLIF) is 0.7% ~ 5% [14, 15].In the course of intraoperative operation, such as the exploration of the pedicle screw tunnel piece outside the pedicle screw insertion process, it is easy to damage the lumbar vertebrae segmental artery during the pedicle screw placement, while it is not easy to solidify it, which can eventually obstruct its purpose of stopping the bleeding. Therefore, persistent active bleeding may occur after PLIF, eventually causing psoas hematoma.

Here we reported a case of a patient who suffered from hemorrhage abdominal pain and bloating after surgery. It is very challenging to detect lower lumbar segmental arterial injury intraoperatively. If not detected early, it can cause massive bleeding, which can lead to serious consequences. Therefore, an early detection of postoperative bleeding caused by vascular injury is critical for providing timely and efficient treatment.

Our patient was initially suffering from neurostimulation in the right lower limb. As the psoas hematoma increased, the hematoma continued to be compressed and stimulated, and the pain became persistent and more intense. Initially, the lumbar X-ray did not suggest swelling of the psoas, which implied that the L5 or S1 nerve root damage was caused by a poor pedicle screw position. Consequently, abdominal CT indicted the formation of psoas hematoma, and the pedicle screw was neither up nor down; therefore, it was difficult to damage the nerve root. We assume the severe pain in the right lower limb was caused by the psoas hematoma, which was triggered by lower lumbar segmental artery injury. This patient's pectoralis hematoma formation caused severe pain in the right lower limb. We discovered that the hematoma compression stimulation caused the psoas contracture. The hematoma was compressed to the lateral posterior disc and the branches of the spinal nerve, which caused the symptoms of the femoral nerve and sciatic nerve. During the early postoperative period, psoas hematoma should be considered in patients with recurrence of radicular symptoms. Classical manifestations include groin pain and radiculopathy symptoms in the affected nerve root distribution [16, 17]. The low back pain, which was experienced by our patient, suggested that non-intervertebral disc factors outside the lumbar may also stimulate the nerves generating symptoms such as low back pain, which can be confused with the lumbar and leg pain caused by degeneration of the spine.

The final review of abdominal CT indicated a psoas hematoma absorption. In the present case, the segmental artery of lower lumbar was injured, which is very challenging for intraoperative detection and can easily cause persistent active bleeding postoperatively. Therefore, it is often manifested by the formation of psoas hematoma. Nonetheless, it is important for patients to take effective measures to stop bleeding, as well as to detect the bleeding on.

In conclusion, lumbar segment arterial injury is prone to anterior and lateral lumbar fusion. However, this case suggests that segmental arterial injury may also occur during PLIF. Our patient had low blood pressure and a high heart rate due to damaged segmental arteries of lower lumbar. His lower hemoglobin suggested a hemorrhagic shock caused by iatrogenic vascular injury. Unexplained severe pain in the lower limb was most probably caused by nerve root stimulation, which was triggered by the psoas hematoma. The patient received DSA vascular embolization that stopped the lower lumbar segmental artery active bleeding.

## Data Availability

All data generated or analysed which related this case report are included in this published article.

## Abbreviations

PLIF: Posterior lumbar interbody fusion
CT: Computerized tomography
DSA: Digital-subtraction angiography
OLIF: Oblique lumbar interbody fusion
